# Integrated procurement and reprocessing planning for reusable medical devices with a limited shelf life

**DOI:** 10.1007/s10729-024-09664-9

**Published:** 2024-01-25

**Authors:** Steffen Rickers, Florian Sahling

**Affiliations:** 1 Department of Production Management, Leibniz Universität Hannover, Königsworther Platz 1, Hannover, 30167 Germany; 2 RPTU School of Management and Economics, Chair of Production Management, University of Kaiserslautern-Landau (RPTU), Gottlieb-Daimler-Straße, Kaiserslautern, 67663 Germany

**Keywords:** Sterile service department, Material logistics in hospitals, Reusable medical devices, Procurement, Reprocessing, Limited shelf life, Column generation

## Abstract

We present a new model formulation for a multiproduct dynamic order quantity problem with product returns and a reprocessing option. The optimization considers the limited shelf life of sterile medical devices as well as the capacity constraints of reprocessing and sterilization resources. The time-varying demand is known in advance and must be satisfied by purchasing new medical devices or by reprocessing used and expired devices. The objective is to determine a feasible procurement and reprocessing plan that minimizes the incurred costs. The problem is solved in a heuristic manner in two steps. First, we use a Dantzig-Wolfe reformulation of the underlying problem, and a column generation approach is applied to tighten the lower bound. In the next step, the obtained lower bound is transformed into a feasible solution using CPLEX. Our numerical results illustrate the high solution quality of this approach. The comparison with a simulation based on the first-come-first-served principle shows the advantage of integrated planning.

## Highlights

- We present a novel model formulation for a multiproduct dynamic order quantity problem with product returns and a reprocessing option that incorporates the limited shelf life of sterile medical devices and capacity constraints on reprocessing and sterilization resources.
- We propose a two-stage heuristic approach based on Dantzig-Wolfe reformulation and column generation techniques to generate high-quality solutions.
- Our simulation study shows a significant cost reduction compared to the standard first-come-first-served approach. These results highlight the practical advantages of the proposed model.

## Introduction

Currently, hospitals are under considerable cost pressure. Germany changed its care fee system to a fee-for-care system in 2004 which forced German hospitals to institute cost-saving measures and process optimization. Until the end of 2003, a daily rate was paid for each patient, which depended on the treatment costs that were actually incurred. Due to a lack of incentives to reduce costs, a new billing procedure based on diagnosis-related groups (DRGs) was introduced in 2004. Based on the primary diagnosis, patients are assigned to a DRG. For each medical treatment, a uniform flat-rate payment is defined, which represents a fixed price for services that do not depend on the individual patient. Thus, the hospital is only profitable if the actual treatment costs do not exceed the specified fixed price. The DRG system therefore transfers both the cost responsibility and the cost risk directly to hospitals.

Rationalization activities often focus on the surgical area. On the one hand, surgeries are the main source of revenue. On the other hand it accounts for a substantial portion of the hospital’s total costs. Hence, in the literature, many approaches focus on the optimization of this area. However, little attention has been given to the supply of sterile goods. The supply of medical devices is not profitable itself, even though cost reductions in this area would have a profit-increasing effect. Moreover, the supply of these medical devices is of enormous importance for the surgical area. For example, the hygiene scandal at Mannheim University Hospital in October 2014 meant that activity in the entire surgical area nearly stopped since sterile goods were not sufficiently reprocessed. Furthermore, [21] pointed out that the supply of sterile goods is of great importance for the surgical area, since surgical teams assume that the medical devices required for operations are not missing or contaminated to ensure the success of the surgery and avoid endangering the health of the patient.

The task of the sterile service department (SSD) is to supply the surgical area with the required quantity of sterile medical devices on time to ensure that the surgery runs smoothly. This work addresses the logistical processes that are necessary to fulfill this task. The processes involved in supplying sterile goods greatly depend on whether medical devices are intended for single or multiple uses. The handling of medical devices is regulated in the European Union in Regulation 2017/745 on medical devices, cf. [13]. Single-use medical devices are intended to be consumed when used in the operating room. From a logistical point of view, procurement and storage processes are necessary for single-use medical devices; cf. [17]. Although reusable medical devices require additional reprocessing, they are often preferable for economic and ecological reasons. Reprocessing yields an additional backward-oriented material flow of medical devices. Thus, complexity has increased due to this reprocessing option. Procuring and reprocessing are two options for satisfying the demand in the operating room. Furthermore, decisions about the procurement times and quantities directly influence the planning of reprocessing, and vice versa. Hence, the present work concentrates on reusable medical devices. The focus is on the development of an optimization model and a suitable solution approach that can be used to determine procurement and reprocessing plans that minimize the total costs. However, these plans must ensure the timely provision of medical devices. The work presented in this paper is based on considerations first discussed in [31].

The remainder of this paper is structured as follows: Section 2 describes the tasks of the central sterile supply department. These tasks include supplying the surgical area with sterile medical devices and reprocessing these devices after utilization. Section 3 provides an overview of the related literature.

In Section 4, a new model formulation for integrated procurement and reprocessing planning of reusable medical devices with a limited storage time is presented. A column generation approach is proposed in Section 5. Based on the generated test instances, the numerical investigations in Section 6 evaluate the performance of the proposed solution approach in terms of computational effort and solution quality. Finally, in Section 7, the presented results are summarized, and further research directions are described.

## Reprocessing and procurement of reusable medical devices

This work focuses on the provision, reprocessing and procurement of reusable sterile goods in hospitals. Reusable sterile goods are medical devices that are intended by the manufacturer for multiple low-germ and sterile usage. They must be reprocessed by the SSD after use before they can be utilized again.

The demand quantities of medical devices can be derived from the surgery schedule as a result of operational surgery planning, which usually includes a planning horizon of one week, cf. [15]. The aim of operational surgery planning is to assign patients to a specific day of the week with a start time for the operation as well as an operating room and team. A distinction must be made between elective and emergency patients. Unlike emergency patients, elective patients do not have critical injuries or illnesses that require immediate surgical care. Typically, elective patients account for 80 to 90% of the operations in a hospital. Thus, due to the short planning horizon of one week, operations on elective patients can be planned under almost deterministic conditions. However, emergency patients cannot be scheduled. According to [4], hospitals have three options for dealing with medical emergencies in operational surgery planning. First, a separate operating room can be reserved exclusively for emergencies. Second, a portion of the capacity in each operating room could be reserved. Third, a combination could be considered. Analogously, these three options can be applied by the SSD to cope with emergency patients. Additionally, safety stocks for particular medical devices are stipulated by law in some countries. In Germany, for example, the Federal Office of Civil Protection and Disaster Assistance provides an inventory list for stocking specific medical devices (see [5]). This list contains, e.g., scissors, scalpels, and forceps.

Compliance with the surgery schedule requires the timely availability and provision of essential medical devices. Consequently, the surgical area must be closely coordinated with the supply of sterile goods to avoid delays. The core task of the SSD is the timely supplying of operating rooms with sterile medical devices of the required quantity and quality. The main process of supplying reusable medical devices can therefore be divided into the following subprocesses: procurement, storage, provision, transport, reprocessing and disposal. Thus, these processes must be coordinated efficiently.

Since hospitals usually do not produce medical devices, the SSD is also responsible for the procurement of medical devices from external suppliers. According to regulations, medical consumables must be disposed of after use. Thus, the material flow of medical consumables through the hospital is strictly forward oriented and corresponds to a classic supply chain. Reusable medical devices, such as surgical instruments, must also be procured, but they can be used several times. Hence, from the perspective of the SSD, the surgical area is both a consumer of sterile medical devices and a supplier of these products in a nonsterile condition. Hence, a closed-loop supply chain must be considered due to the additional reverse material flow from the operating room to the SSD and the reprocessing option. With a portion of approximately 80% of working time, reprocessing constitute the main tasks of the SSD. The whole process can be described as a reprocessing cycle that is identical for all reusable medical devices. This reprocessing cycle starts with the usage of medical devices in operating rooms. Medical devices are typically provided in surgery-specific sets that must be extracted before usage. Afterwards, the utilized medical devices are returned to the SSD. It is worth mentioning that even the medical devices that were not used must be reprocessed if their packaging was opened or damaged.

At the beginning of a reprocessing operation, medical devices are precleaned to remove contamination. Additionally, a prescreening of medical devices with uneven surfaces is necessary because contamination is difficult to remove. Damaged medical devices are disposed of and thus leave the reprocessing cycle directly. The decontamination of medical devices includes cleaning, disinfection, rinsing and drying. For this process, medical devices are placed in sieves. These sieves are loaded into washer-disinfectors. Thermal disinfection is based on the so-called A_0_ concept. The A_0_ value describes the time duration required to kill microorganisms at a given temperature. The required A_0_ value depends on the risk classification of a medical device that was introduced by [38] who classified medical devices as noncritical, semicritical or critical based on the risk of infection related to the usage on the patient. Theoretically, each medical device can be reprocessed by any time-temperature combination in which the corresponding A_0_ value is at least as high as the device-specific value. For example, an A_0_ value of 600 can be achieved by “600 seconds at 80^∘^ C” or by “60 seconds at 90^∘^ C”. However, the temperature must not exceed the device-specific temperature tolerance stipulated by its manufacturer. The final rinsing and drying guarantee that no residue of the chemicals used for cleaning remains on the medical devices.

Afterwards, the cleaning results are controlled, and the functionality is tested. The medical devices are packed into surgery-specific sets. Subsequent sterilization is used to kill the remaining microorganisms. Various sterilization types as well as different time-temperature combinations are available. The sterilization procedure is selected based on the requirements of the medical device. Thermostable medical devices are usually sterilized by steam or hot air. For heat-sensitive medical devices, different methods are available with lower process temperatures. Notably, the complete reprocessing operation includes precleaning, decontamination and sterilization.

If the medical devices are not provided directly in operating rooms, they can be stored unprotected on shelves or protected in cabinets or drawers. However, the shelf life of sterilized medical devices depends on the type of packaging and storage conditions. If these devices are stored unprotected, they must be used within 48 hours. If they are stored protected, the shelf life can be up to 12 months. The storage space for sterile medical devices is usually limited since the SSD is often located close to the operating rooms; long transportation may increase the risk of contamination. Withdrawal from storage is based on the first-in–first-out principle to avoid exceeding the maximum storage time. If the maximum storage time is exceeded, the medical devices must be reprocessed again.

## Related work

The handling of medical devices in hospitals has received little attention in literature. An overview of the logistics of sterile medical devices can be found in [45]. Most scientific publications examining the reprocessing of medical devices describe rules and legal requirements for reprocessing and sterilization. Insights are given, for example, by [22] and [33].

In the literature, there are a few approaches to operating room planning that include the availability of medical devices or their reprocessing. Meskens et al. [24] presented an optimization problem for the generation of a surgery schedule, in which reprocessable medical devices are considered renewable resources. Each type of operation requires a characteristic number of different medical devices that are only available in limited quantities. Guinet and Chaabane [14, 44] and [43] consider the resource limitations of medical devices when preparing surgery schedules.

Cardoen et al. [6, 7] include the necessary reprocessing time of medical devices in operating room planning. After use, medical devices are not available for a fixed number of periods, so operations of the same type cannot immediately follow one another. Al Hasan et al. [3] also created a surgery schedule taking into account the availability of medical devices and the reprocessing time. To comply with the surgery schedule, a medical device can be prioritized for reprocessing, leading to additional costs.

Coban [8] formulated a mixed-integer model to plan surgeries and reprocess medical devices in an integrated manner. However, only homogeneous medical devices are considered in this model. A certain number of sterile medical devices are provided for each operation. If the number of sterile medical devices is not sufficient, the operation cannot take place and must be postponed. It is not possible to order missing medical devices to comply with the surgical plan. After use, the medical devices can be reprocessed and stored. However, only one type of sterilization is considered.

Most of the literature on inventory management for medical devices addresses medical consumables. Ahmadi et al. [1] and [34] provide an overview. In the literature on inventory management of reusable medical devices, reprocessing plays a rather secondary role. One reason is that reprocessing is outsourced to an external service provider and is therefore no longer part of the planning problem.

In [42], an external service provider conducts the sterilization of medical devices. The authors consider an integrated lot sizing and transportation problem with deterministic demand to determine the optimal order times and quantities. Diamant et al. [11] also assume that sterilization is outsourced. The authors determine the minimum quantities required to ensure a defined service level for stochastic demand.

The majority of publications on reprocessing medical devices deal with either the rules to be observed in reprocessing or the reliability of the overall process. Several publications focus on decontamination resources, which, according to [10], are the bottleneck of the entire reprocessing cycle. Ozturk et al. [28] developed a mixed-integer linear program to model the decontamination step as a batch scheduling problem with multiple identical machines. Before decontamination, the incoming medical devices are grouped into a batch and assigned to a machine. The earliest possible planning time is obtained for each medical device. The authors aim to minimize the total reprocessing time. Ozturk et al. [27] develop a problem-specific branch & bound heuristic to solve larger test instances. Xu and Wang [47] generalize the problem presented by [28] to the case of nonidentical machines with different capacities. Furthermore, [26] examines a special case in which an external service provider conducts sterilization. The used medical devices are collected after the operation and sent to the service provider.

The work of [40, 41] and [36] addresses the question of whether medical devices should be sterilized in a centralized or decentralized manner. However, the question of centralized or decentralized reprocessing and the purchasing of reprocessing resources is largely a strategic decision.

Lot sizing problems are related to order quantity planning. There are numerous approaches in the literature that take perishability or limited shelf life into account, e.g., [19] and [29]. In addition, there are approaches in which limited storage capacity is considered in planning. See [12] and [20] for the one-product case and [2, 23, 25] and [46] for the multiproduct case. Numerous approaches take a remanufacturing option into account when planning lot sizes. See [39] and [37] for the one-product case without capacity restrictions. Approaches for capacity-restricted lot sizing with remanufacturing are considered, for example, by [30, 35] and [9].

To fully map the planning situation in the supply of sterile goods, procurement and reprocessing activities must be planned simultaneously by considering reprocessing and storage capacities and the limited storage time of medical devices. However, the publications presented above cover only a few aspects of these requirements. To the best of our knowledge, publications that incorporate procurement, reprocessing and the limited shelf life of medical devices do not exist.

## The integrated procurement and reprocessing planning problem for reusable medical devices

### Model assumptions

**Fig. 1:**
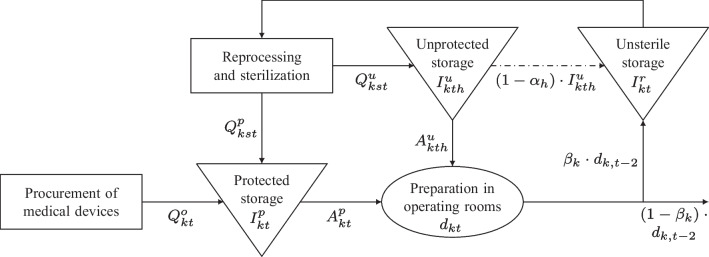
Material flow of reusable medical devices

In the integrated *Procurement and Reprocessing Planning Problem* (PRPP), the planning horizon is divided into *T* discrete periods $(t\in \mathcal {T})$. Typically, the length of the planning horizon depends on the surgery schedule. Consequently, a planning horizon of one week is often assumed. We consider a seven-day week where each day consists of two shifts with a length of 8 hours, i.e., each period *t* equals one shift. *K* different medical devices $(k \in \mathcal {K})$ can be procured or reprocessed. In Fig. 1, cf. [31], the material flow of reusable medical devices is described.

### Procurement of medical devices

Medical devices can be procured from an external supplier. For better differentiation, the notation related to the procurement process has been marked with superscript *o*. Variable material costs $pc^o_k$ are incurred for each ordered quantity $Q^o_{kt}$ of medical device *k* in period *t*. In addition, each order of medical device *k* incurs quantity-independent ordering costs $oc^o_k$. The binary variable $\gamma ^o_{kt}$ equals 1 if medical device *k* is ordered in period *t*. Otherwise, this variable $\gamma ^o_{kt}$ is equal to 0. Notably, the procured medical devices are delivered in sterile and protected conditions. Furthermore, they can be used or stored directly without delay.

### Reprocessing of medical devices

The complete reprocessing operation includes the steps of precleaning, decontamination and sterilization, as described in Section 2. However, [26] noted that decontamination resources, i.e., the washer-disinfectors for cleaning, disinfection, rinsing and drying, are of particular importance, as they often represent a bottleneck in terms of time in the reprocessing cycle. Thus, it is sufficient to focus on decontamination resources.

Different reprocessing types $(s \in \mathcal {S}=\{1,\dots ,S\})$ can be identified from the existing time-temperature combinations (cf. Section 2). However, due to different temperature tolerances, not every time-temperature combination is permissible for each medical device, e.g., thermolabile medical devices cannot be reprocessed at 90^∘^ C. Thus, the set $\mathcal {K}_s$ includes those medical devices that can be reprocessed by type *s*. The subset $\mathcal {S}_k$, on the other hand, includes those types through which medical device *k* can be reprocessed. It is worth mentioning that a reprocessing operation does not necessarily require homogeneous medical devices; rather, different medical devices can be reprocessed at the same time.

The integer decision variable $\chi ^r_{st}$ denotes the number of reprocessing operations of type *s* carried out in period *t*. Here, the notation related to reprocessing has been marked with superscript *r*. The duration of a reprocessing operation of type *s* is described by $ts_s^r$, which depends neither on the assigned medical devices nor on the reprocessing quantity. The capacity $c^r_t$ limits the number of reprocessing operations that can be carried out in period *t* can be deleted. The fixed costs $sc_s^r$ are incurred for each reprocessing operation of type *s*. Notably, because of the higher energy consumption of heating, high-temperature reprocessing, although shorter in duration, is more costly than time-temperature combinations with lower temperatures but longer durations. Furthermore, the space $vol^{\max }$ of a reprocessing operation that is consumed by medical devices *k* with space requirements $vol_k$ is also limited.

The decision variable $Q^p_{kst}$ specifies the reprocessed quantity of medical device *k* in period *t* using type *s*, which is stored protected e.g. in cabinets or drawers afterwards (denoted by superscript *p*). The packaging of one unit of medical device *k* incurs packaging costs $pc^p_k$ if stored protected. Analogously, $Q^u_{kst}$ specifies the reprocessed quantity of medical device *k* in period *t* using type *s* that is stored unprotected e.g. on shelves afterwards (denoted by superscript *u*). Packaging costs $pc^u_{k}$ also apply for each unit if stored unprotected. Since protected storage requires additional packaging compared with that of unprotected storage, the packaging costs for protected storage are higher $(pc^p_{k}>pc^u_{k}\,\,\forall \, k\in \mathcal {K})$. The reprocessed medical devices can be used to fulfill the demand at the end of a period.

### Shelf life and storage of medical devices

For protected stored medical devices $I^p_{kt}$, the maximum shelf life clearly exceeds the length of the planning horizon, so the shelf life can be neglected in this case. However, for unprotected storage, the storage duration must be monitored explicitly. The index $h \in \mathcal {H}=\lbrace 0,\dots ,h^{\max },h^{\max }+1\rbrace$ describes the number of periods for which the medical devices have already been stored unprotected. After $h^{\max }+1$ periods of unprotected storage, a medical device exceeds the maximum shelf life and will lose its sterile condition. The parameter $\alpha _h$ describes the state of sterility in the storage period *h*. For storage period $h\le h^{\max }$, the parameter $\alpha _h$ is equal to 1. Otherwise, the parameter $\alpha _h$ equals 0 $(\alpha _h=0\,\, \forall \,\, h> h^{\max })$.

The integer decision variable $I^u_{kth}$ indicates the inventory of the unprotected stored medical device *k* at the end of period *t* in storage period *h*. The decision variable $I^p_{kt}$ describes the stock of protected stored medical devices *k* at the end of period *t*. Furthermore, the inventory of used and unsterile medical devices *k* at the end of period *t* is denoted by $I_{kt}^r$.

In each period *t*, the inventory of unprotected and protected stored sterile medical devices is limited by the capacity limit $c^{I^u}$ or $c^{I^p}$. The parameter $vol_k$ indicates the storage space requirement for one unit of medical device *k*. However, the storage capacity for used medical devices is assumed to be unlimited.

### Demand fulfillment and returns

The dynamic demand $d_{kt}$ of medical device *k* in period *t* is derived from the surgery schedule. Thus, it is assumed that the period-specific demand $d_{kt}$ is known in advance and must be completely satisfied. The requirements of medical device *k* in period *t* can be covered by both protected and unprotected stored medical devices. The respective withdrawal quantities from storage are referred to as staging quantities. The decision variable $A^u_{kth}$ corresponds to the staging quantity of unprotected stored medical device *k* in period *t* with storage duration *h*. Furthermore, $A^p_{kt}$ denotes the staging quantity of protected stored medical device *k* in period *t*.

After utilization, a portion $0 \le \beta _k \le 1$ of medical device *k* returns to the depot of used medical devices in period *t*. However, a time delay of two periods is assumed, so the returns $r_{kt}$ of medical device *k* can be determined by $r_{kt}=\lfloor \beta _k \cdot d_{k,t-2} \rfloor$. Due to damage or signs of aging, the nonreturning portion $(1-\beta _k)$ of medical device *k* cannot be reprocessed and must be disposed of.

The goal of the PRPP is to determine a feasible procurement and reprocessing plan that completely satisfies the derived demand and minimizes the procurement and reprocessing costs.

### Mathematical model formulation

Using the notation presented in Table 1, the integrated procurement and reprocessing planning problem for reusable medical devices can be mathematically modeled as follows:

**Table 1:** Notation used for the PRPP

Indices and index sets:	
$h \in \mathcal {H}$	set of storage periods $(h \in \{0,\ldots ,h^{\max },h^{\max }+1\})$
$k \in \mathcal {K}$	set of medical devices $(k \in \{1,\ldots ,K\})$
$s \in \mathcal {S}$	set of types $(s \in \{1,\ldots ,S\})$
$t \in \mathcal {T}$	set of periods $(t \in \{1,\ldots ,T\})$
$k \in \mathcal {K}_s \subseteq \mathcal {K}$	subset of medical devices requiring type *s*
$s \in \mathcal {S}_k \subseteq \mathcal {S}$	subset of types that can reprocess medical device *k*
Parameters:	
$\alpha _{h}$	shelf life indicator in storage period *h*
$\beta _{k}$	portion of reprocessable medical device *k* after utilization
$bigM_{kt}$	sufficiently large number for medical device *k* in period *t*
$c^{I^u}$	storage capacity for unprotected stored medical devices
$c^{I^p}$	storage capacity for protected stored medical devices
$c^r_{t}$	capacity of reprocessing resources in period *t*
$d_{kt}$	demand of medical device *k* in period *t*
$oc^o_k$	fixed procurement costs per order of medical device *k*
$pc^o_{k}$	variable procurement costs per unit of medical device *k*
$pc^p_{k}$	variable packaging costs for one protected stored unit of medical device *k*
$pc^u_{k}$	variable packaging costs for one unprotected stored unit of medical device *k*
$r_{kt}$	returns of medical device *k* in period *t*
$sc^r_{s}$	fixed costs for a reprocessing operation of type *s*
$ts^r_{s}$	duration of a reprocessing operation of type *s*
$vol_{k}$	space requirement for reprocessing or storing one unit of medical device *k*
$vol^{\max }$	space capacity for reprocessing
Decision variables:	
$A^p_{kt} \in \mathbb {N}_0$	staging quantity of protected stored medical device *k* in period *t*
$A^u_{kth}\in \mathbb {N}_0$	staging quantity of unprotected stored medical device *k* in period *t* and storage period *h*
$I^p_{kt}\in \mathbb {N}_0$	protected end-of-period inventory of sterile medical device *k* in period *t*
$I^r_{kt}\in \mathbb {N}_0$	end-of-period inventory of used medical device *k* in period *t*
$I^u_{kth}\in \mathbb {N}_0$	unprotected end-of-period inventory of sterile medical device *k* in period *t* and storage period *h*
$Q^o_{kt}\in \mathbb {N}_0$	ordered quantity of medical device *k* in period *t*
$Q^{p}_{kst}\in \mathbb {N}_0$	reprocessing quantity of medical device *k* with type *s* in period *t* with subsequent protected storage
$Q^{u}_{kst}\in \mathbb {N}_0$	reprocessing quantity of medical device *k* with type *s* in period *t* with subsequent unprotected storage
$\gamma ^o_{kt}\in \{0,1\}$	binary ordering variable for medical device *k* in period *t*
$\chi ^r_{st}\in \mathbb {N}_0$	number of reprocessing operations of type *s* in period *t*

## Model PRPP

1 $$\begin{aligned} \min \, Z =&\sum _{k \in \mathcal {K}} \sum _{t \in \mathcal {T}}\left( oc^o_k\cdot \gamma ^o_{kt} + pc^o_{k}\cdot Q^o_{kt} \right) \!+ \!\sum _{s \in \mathcal {S}} \sum _{t \in \mathcal {T}} sc^r_{s} \cdot \chi ^r_{st} \nonumber \\&+ \sum _{k \in \mathcal {K}} \sum _{s \in \mathcal {S}_k} \sum _{t \in \mathcal {T}} \left( pc^u_{k}\cdot Q^{u}_{kst} + pc^p_{k} \cdot Q^{p}_{kst} \right) \end{aligned}$$

subject to

2 $$\begin{aligned} A^p_{kt} + \sum _{h=0}^{h^{\max }} A^u_{kth} = d_{kt} \forall \, k \in \mathcal {K},t\in \mathcal {T} \end{aligned}$$

3 $$\begin{aligned} \sum _{s \in \mathcal {S}_k} Q^{u}_{kst} - A^u_{kt0} = I^u_{kt0} \forall \, k \in \mathcal {K},t\in \mathcal {T} \end{aligned}$$

4 $$\begin{aligned} \alpha _{h} \cdot I^u_{k,t-1,h-1} - A^u_{kth} \!=\! I^u_{kth} \!\!\qquad \qquad \!\!\!\!\forall \, k \in \mathcal {K},t\in \mathcal {T},h\in \mathcal {H}\backslash \{0\} \end{aligned}$$

5 $$\begin{aligned} I^p_{k,t-1} + Q^o_{kt} + \sum _{s \in \mathcal {S}_k} Q^{p}_{kst} - A^p_{kt} = I^p_{kt} \qquad \qquad \forall \, k \in \mathcal {K},t\in \mathcal {T} \end{aligned}$$

$$\begin{aligned} I^r_{k,t-1} + r_{kt} + \sum _{h=1}^{h^{\max }+1} (1- \alpha _{h}) \cdot I^u_{k,t-1,h-1} - \sum _{s \in \mathcal {S}_k} \left( Q^{p}_{kst} + Q^{u}_{kst} \right) = I^r_{kt} \end{aligned}$$

6 $$\begin{aligned} \forall \, k \in \mathcal {K},t\in \mathcal {T} \end{aligned}$$

7 $$\begin{aligned} Q^o_{kt} \le bigM_{kt} \cdot \gamma ^o_{kt} \forall \, k \in \mathcal {K},t\in \mathcal {T} \end{aligned}$$

8 $$\begin{aligned} \sum _{k \in \mathcal {K}_s} vol_k \cdot \left( Q^{p}_{kst} + Q^{u}_{kst} \right) \le vol^{\max } \cdot \chi ^r_{st} \forall \, s\in \mathcal {S},t\in \mathcal {T} \end{aligned}$$

9 $$\begin{aligned} \sum _{s \in \mathcal {S}} ts^r_{s} \cdot \chi ^r_{st} \le c^r_{t} \forall \, t\in \mathcal {T} \end{aligned}$$

10 $$\begin{aligned} \sum _{k \in \mathcal {K}} \sum _{h=0}^{h^{\max }} vol_k \cdot I^u_{kth} \le c^{I^u} \forall \, t\in \mathcal {T} \end{aligned}$$

11 $$\begin{aligned} \sum _{k \in \mathcal {K}} vol_k \cdot I^p_{kt} \le c^{I^p} \forall \, t\in \mathcal {T} \end{aligned}$$

12 $$\begin{aligned} A^p_{kt},\,A^u_{kth} \in \mathbb {N}_0 \forall \, k \in \mathcal {K},t\in \mathcal {T}, h \in \mathcal {H} \end{aligned}$$

13 $$\begin{aligned} I^p_{kt},\,I^r_{kt},\,I^u_{kth} \in \mathbb {N}_0 \forall \, k \in \mathcal {K},t\in \mathcal {T}, h \in \mathcal {H} \end{aligned}$$

14 $$\begin{aligned} Q^o_{kt},\,Q^{p}_{kst},\,Q^{u}_{kst} \in \mathbb {N}_0 \forall \, k \in \mathcal {K},s \in \mathcal {S}_k,t\in \mathcal {T} \end{aligned}$$

15 $$\begin{aligned} \chi ^r_{st} \in \mathbb {N}_0 \forall \, s \in \mathcal {S},t\in \mathcal {T} \end{aligned}$$

16 $$\begin{aligned} \gamma ^o_{kt}\in \{0,1\} \forall \, k \in \mathcal {K},t\in \mathcal {T} \end{aligned}$$

The inventory balance constraints are represented by (2) to (6). Equations (2) ensure that the given demand $d_{kt}$ is fulfilled completely by the cumulative staging quantities for each medical device *k* in period *t*. According to constraints (3), the inventory of unprotected medical devices with storage time $h=0$ only consists of the directly reprocessed quantities in the considered period *t*, unless the devices are directly used for demand fulfillment. Equations (4) represent the inventory balance constraints for unprotected stored sterile medical devices with storage time $h\ge 1$. However, these restrictions also ensure that medical devices that reach the maximum storage time $h=h^{\max }+1$ in period *t* will lose their sterile condition. Equations (5) represent the inventory balance constraints for protected stored medical devices. Constraints (6) describe the inventory balance equations for nonsterile medical devices, including medical devices with an expired storage time.

The constraints (7) link the integer variables for procurement $Q^o_{kt}$ with the binary variables $\gamma ^o_{kt}$. If medical device *k* is ordered in period *t* $(Q^o_{kt}>0)$, an ordering process is needed. This forces the binary order variable $\gamma ^o_{kt}$ to the value one. The parameter $bigM_{kt}$ represents a sufficiently large number and is defined as follows:

17 $$\begin{aligned} bigM_{kt} = \sum _{\tau =t}^{T} d_{k\tau } \forall \, k \in \mathcal {K},t\in \mathcal {T}. \end{aligned}$$

Constraints (8) combine the reprocessing quantities $Q^{p}_{kst}$ and $Q^{u}_{kst}$ with the number of reprocessing operations $\chi ^r_{st}$. If at least one medical device *k* is reprocessed in period *t* with type *s*, i.e., $\sum _k \left( Q^{p}_{kst} + Q^{u}_{kst} \right) > 0$, the integer variable $\chi ^r_{st}$ equals the required number of reprocessing operations. The capacity constraints (9) ensure that the given capacity of the reprocessing resource is not exceeded; i.e., the maximum number of reprocessing operations that can be carried out in period *t* is limited. Constraints (10) and (11) restrict the storage capacities of protected and unprotected stored sterile medical devices. Constraints (14) to (16) define the dimensions of the decision variables.

If the reprocessing capacity $c^r_t$ is set to zero for all periods, medical devices cannot be reprocessed and the complete period-specific demand $d_{kt}$ for medical device *k* must be satisfied by procurement. In this case, the PRPP corresponds to an uncapacitated lot sizing problem with inventory bounds. Since this lot sizing problem is proven to be $\mathcal{N}\mathcal{P}$-hard (see [2]), the PRPP is also $\mathcal{N}\mathcal{P}$-hard. Due to the $\mathcal{N}\mathcal{P}$-hardness of the PRPP, the computational effort to solve this problem optimally using a standard MILP solver is usually prohibitively large for all but tiny problem instances. Thus, a heuristic is required to determine an appropriate solution within a reasonable time frame.

## A solution approach based on column generation

### Idea of Dantzig-Wolfe decomposition and column generation

The proposed solution approach is based on Dantzig-Wolfe decomposition, which is used to reformulate the PRPP. The PRPP is decomposed into a master problem denoted as MP-PRPP and *K* device-specific subproblems denoted as SP-PRPP_k_. A column generation (CG) approach is applied to solve the master problem. The master problem is initialized with a small number of columns. In an iterative procedure, the subproblems are solved to generate new columns for the master problem. If a new column will lead to a reduction in the objective function value of the master problem, it is incorporated into the master problem. However, the column generation approach terminates if no further columns can be generated that reduce the current objective function value of the master problem. The process of the CG approach for the PRPP is described below. Further implementation details of the solution approach can be found in [31].

### The master problem

From a mathematical perspective, the master problem corresponds to a set partitioning reformulation of the PRPP. The objective of the *Set Partitioning Problem (SPP)* is to select exactly one procurement and reprocessing plan for each medical device *k* at a minimal total cost. The selection of plans must meet the capacity restrictions for protected and unprotected storage as well as for reprocessing.

First, it is assumed that all possible and feasible procurement and reprocessing plans $\mathcal {N}_k$ are known for medical device *k* in advance. A procurement and reprocessing plan is feasible if the demand is met in each period. A procurement and reprocessing plan *n* is described by procurement quantities $\overline{Q}_{kt}^{o(n)}$ and decisions $\overline{\gamma }_{kt}^{o(n)}$. In addition, each plan *n* provides information regarding the quantities of protected and unprotected reprocessed medical devices *k* of type *s* in period *t*, which are described by parameters $\overline{Q}^{p(n)}_{kst}$ and $\overline{Q}^{u(n)}_{kst}$. Furthermore, each plan *n* contains the end-of-period inventory of protected $\overline{I}^{p(n)}_{kt}$ and unprotected $\overline{I}^{u(n)}_{kth}$ stored medical devices *k* in period *t*, where the storage duration *h* is also known.

The fixed and variable procurement costs of medical device *k* in plan *n* can be determined with respect to $\overline{\gamma }_{kt}^{o(n)}$ and $\overline{Q}_{kt}^{o(n)}$. For plan *n*, the variable packaging costs of medical device *k* can be calculated using the parameters $\overline{Q}^{p(n)}_{kst}$ and $\overline{Q}^{u(n)}_{kst}$. However, the reprocessing costs depend on the selected plans and the number of reprocessing operations $\chi _{st}^r$. Thus, these costs must be implicitly taken into account in the objective function of the master problem.

The parameters $\overline{Q}^{p(n)}_{kst}$ and $\overline{Q}^{u(n)}_{kst}$ allow for the determination of the capacity requirements for reprocessing medical device *k* using type *s* in period *t* with plan *n*. In addition, the required storage capacity for protected or unprotected storage can be derived with respect to $\overline{I}^{p(n)}_{kt}$ and $\overline{I}^{u(n)}_{kth}$ for medical device *k* in period *t* with plan *n*.

For the selection of a plan *n* for medical device *k*, the binary variable $\vartheta _{kn}$ is used, which is defined as follows:

18 $$\begin{aligned} \vartheta _{kn}=\left\{ \begin{array}{ll} 1, \, \text {if plan~n}\in \mathcal {N}_k \text {is selected for medical device~k}\\ 0, \, \text {otherwise.} \end{array}\right. \end{aligned}$$

The objective of the master problem is to select exactly one plan for each medical device *k* so that the total procurement and reprocessing costs are minimized and the capacity restrictions are met.

The model formulation of the master problem is introduced using the additional notation in Table 2.

**Table 2:** Additional notation used for master problem MP-PRPP

Indices and index sets:	
$n \in \mathcal {N}_k$	set of procurement and reprocessing plans for medical device *k*
Parameters:	
$\overline{I}^{p(n)}_{kt}$	protected end-of-period inventory of sterile medical device *k* in period *t* of plan *n*
$\overline{I}^{u(n)}_{kth}$	unprotected end-of-period inventory of sterile medical device *k* in period *t* of plan *n*
$\overline{Q}^{o(n)}_{kt}$	ordered quantity of medical device *k* in period *t* of plan *n*
$\overline{Q}^{p(n)}_{kst}$	reprocessing quantity of medical device *k* in period *t* of type *s* with subsequent protected storage in plan *n*
$\overline{Q}^{u(n)}_{kst}$	reprocessing quantity of medical device *k* in period *t* of type *s* with subsequent unprotected storage in plan *n*
$\overline{\gamma }^{o(n)}_{kt}$	level of the binary ordering variable of medical device *k* in period *t* in plan *n*
Decision variables:	
$\vartheta _{kn} \in \{0,1\}$	binary selection variable for plan *n* of medical device *k*
Dual variables:	
$\pi ^{r}_{st}\in \mathbb {R}$	dual variable for reprocessing capacity constraints of type *s* in period *t*
$\pi ^{I^p}_{t}\in \mathbb {R}$	dual variable for protected storage capacity constraints in period *t*
$\pi ^{I^u}_{t}\in \mathbb {R}$	dual variable for unprotected storage capacity constraints in period *t*
$\sigma _k \in \mathbb {R}$	dual variable for convexity constraints of medical device *k*

**Model MP-PRPP**

19 $$\begin{aligned} \min Z =&\sum _{k \in \mathcal {K}} \sum _{n \in \mathcal {N}_k} \sum _{t \in \mathcal {T}} \left( pc^o_{k}\cdot \overline{Q}^{o(n)}_{kt} + oc^o_k\cdot \overline{\gamma }^{o(n)}_{kt} \right) \cdot \vartheta _{kn} \nonumber \\&+ \!\sum _{k \in \mathcal {K}} \!\sum _{n \in \mathcal {N}_k} \sum _{s \in \mathcal {S}_k} \sum _{t \in \mathcal {T}} \!\left( pc^p_{k}\!\cdot \! \overline{Q}^{p(n)}_{kst} \!+\! pc^u_{k}\!\cdot \! \overline{Q}^{u(n)}_{kst} \right) \!\cdot \! \vartheta _{kn} \nonumber \\&+ \sum _{s \in \mathcal {S}} \sum _{t \in \mathcal {T}} sc^r_{s} \cdot \chi ^r_{st} \end{aligned}$$

subject to dual variables

$$\begin{aligned} \sum _{k \in \mathcal {K}_s} \sum _{n \in \mathcal {N}_k} vol_k \cdot \left( \overline{Q}^{p(n)}_{kst} + \overline{Q}^{u(n)}_{kst}\right) \cdot \vartheta _{kn} \le vol^{\max } \cdot \chi ^r_{st} \end{aligned}$$

20 $$\begin{aligned} \quad \forall \, s \in \mathcal {S},t\in \mathcal {T} \quad \rightarrow \quad \pi ^{r}_{st} \end{aligned}$$

21 $$\begin{aligned} \sum _{s \in \mathcal {S}} ts^r_{s} \cdot \chi ^r_{st} \le c^r_{t} \forall \, t\in \mathcal {T} \quad \rightarrow \quad \pi ^{r}_{st} \end{aligned}$$

22 $$\begin{aligned} \sum _{k \in \mathcal {K}}\! \sum _{n \in \mathcal {N}_k} \left( \sum _{h=0}^{h^{\max }} vol_k \!\cdot \! \overline{I}^{u(n)}_{kth} \right) \cdot \vartheta _{kn} \le c^{I^u} \!\!\!\qquad \forall \!\, t\in \mathcal {T} \quad \rightarrow \!\!\quad \pi ^{I^u}_t \end{aligned}$$

23 $$\begin{aligned} \sum _{k \in \mathcal {K}} \sum _{n \in \mathcal {N}_k} \left( vol_k \cdot \overline{I}^{p(n)}_{kt} \right) \cdot \vartheta _{kn} \le c^{I^p} \qquad \forall \, t\in \mathcal {T} \quad \rightarrow \quad \pi ^{I^p}_t \end{aligned}$$

24 $$\begin{aligned} \sum _{n \in \mathcal {N}_k} \vartheta _{kn} \ge 1 \forall k \in \mathcal {K} \quad \rightarrow \quad \sigma ^{{r}}_k \end{aligned}$$

25 $$\begin{aligned} \vartheta _{kn} \in \{0,1\} \forall \, k \in \mathcal {K},n \in \mathcal {N}_k \quad \rightarrow \quad \pi ^r_t \end{aligned}$$

26 $$\begin{aligned} \chi ^r_{st} \in \mathbb {N}_0^+ \forall \, s\in \mathcal {S},t\in \mathcal {T} \quad \rightarrow \quad \pi ^r_t \end{aligned}$$

The objective function (19) minimizes the total costs. These costs include variable ($pc^o_{k}\cdot \overline{Q}^{o(n)}_{kt}$) and fixed procurement costs ($oc^o_k\cdot \overline{\gamma }^{o(n)}_{kt}$), variable packaging costs ($pc^p_{k}\cdot \overline{Q}^{p(n)}_{kst} + pc^u_{k}\cdot \overline{Q}^{u(n)}_{kst}$) each associated with the selected plans and fixed reprocessing costs.

The inequalities (20) link the selection variables $\vartheta _{kn}$ with the number of performed reprocessing operations $\chi ^r_{st}$. If a plan *n* is selected $(\vartheta _{kn}>0)$ with positive reprocessing quantities $(\overline{Q}^{p(n)}_{kst} + \overline{Q}^{u(n)}_{kst}>0)$, at least one reprocessing operation is needed, i.e., $\chi ^r_{st}>0$. The dual variables $\pi ^{r}_{st}$ correspond to these restrictions. The capacity restrictions (21) guarantee that the reprocessing capacity $c_t^r$ is not exceeded. The corresponding dual variables are denoted by $\pi ^{r}_{ st}$.

Constraints (22) and (23) reflect the capacity limitations for unprotected and protected storage. The corresponding dual variables are denoted by $\pi ^{I^u}_t$ and $\pi ^{I^p}_t$.

The inequalities (24) ensure that at least one plan is selected for each medical device *k*. Theoretically, more than one plan can be selected. However, due to the minimization of the objective function, exactly one plan must be selected because additional plans lead to additional costs. The dual variables of these restrictions are denoted by $\sigma _k$.

It is worth mentioning that if all procurement and reprocessing plans are known in advance, the optimal solution of the SPP equals the optimal solution of the PRPP. However, since the number of plans increases exponentially, the determination of all plans in advance would be very time-consuming. Thus, the master problem is solved by a column generation approach, where new plans are determined iteratively by solving medical device-specific subproblems. These new plans are subsequently included in the master problem. A new procurement and reprocessing plan *n* is included in the master problem, i.e., in the set $\mathcal {N}_k$, if it yields a reduction in the current objective function value, i.e., if the reduced costs are negative. However, a rising number of plans will lead to a substantial increase in the numerical effort to solve the master problem to optimality. Thus, only the LP relaxation of the master problem is solved, i.e., $\vartheta _{kn} \in [0,1]$ and $\chi ^r_{st} \in \mathbb {R}_0^+$. Now, convex combinations of procurement and reprocessing plans are allowed according to (24). In general, the column generation approach does not terminate with an integer solution, but provides a lower bound that can be used to determine a feasible upper bound as described below.

### The medical device-specific subproblem

The objective of the medical device-specific subproblem SP-PRPP_k_ is to generate new procurement and reprocessing plans for each medical device *k*. The notation of the medical device-specific subproblem SP-PRPP_k_ is known from Table 1, additional notation is provided in Table 3.

In each iteration of the CG approach, a new plan is added to the relaxed master problem. The master problem is solved again, and the dual variables $\overline{\sigma }_k$, $\pi ^r_{st}$, $\pi ^{I^u}_{t}$ and $\pi ^{I^p}_{t}$ are updated and incorporated into the objective function of each subproblem as parameters $\overline{\sigma }_k$, $\overline{\pi }^r_{st}$, $\overline{\pi }^{I^u}_{t}$ and $\overline{\pi }^{I^p}_{t}$.

**Table 3:** Additional notation for medical device-specific subproblem SP-PRPP_k_

Parameters:	
$\overline{\pi }^{r}_{st}$	level of the dual variable $\pi ^{r}_{st}$ corresponding to the reprocessing capacity restriction of type *s* in period *t*
$\overline{\pi }^{I^p}_{t}$	level of the dual variable $\pi ^{I^p}_{t}$ corresponding to the protected storage capacity restriction in period *t*
$\overline{\pi }^{I^u}_{t}$	level of the dual variable $\pi ^{I^u}_{t}$ corresponding to the unprotected storage capacity restriction in period *t*
$\overline{\sigma }_k$	level of the dual variable $\sigma _k$ corresponding to the convexity constraints of medical device *k*

**Model SP-PRPP**_k_ **of medical device** *k*

27 $$\begin{aligned} \min \, Z_k^{SP} =&\sum _{t \in \mathcal {T}}\left( pc^o_{k}\cdot Q^o_{kt} + oc^o_k\cdot \gamma ^o_{kt} \right) \nonumber \\ +&\sum _{s \in \mathcal {S}_k} \sum _{t \in \mathcal {T}} \left( pc^p_{k}\cdot Q^p_{kst} + pc^u_{k}\cdot Q^u_{kst}\right) \nonumber \\ -&\sum _{s \in \mathcal {S}_k} \sum _{t \in \mathcal {T}} \overline{\pi }^{r}_{st} \cdot vol_k \cdot \left( Q^p_{kst} + Q^u_{kst} \right) \nonumber \\ -&\sum _{t \in \mathcal {T}} \sum _{h=0}^{h^{\max }} \overline{\pi }^{I^u}_{t} \!\cdot \! vol_k \cdot \! I^u_{kth}\!-\! \sum _{t \in \mathcal {T}} \overline{\pi }^{I^p}_{t} \!\cdot vol_k \!\cdot \! I^p_{kt} \!-\! \overline{\sigma }_k \end{aligned}$$

subject to

\ref {Eq:ModspsDemFullf}$_{k}$ $$\begin{aligned} 2_{k} A^p_{kt} + \sum _{h=0}^{h^{\max }} A^u_{kth} = d_{kt} \forall \, t\in \mathcal {T} \end{aligned}$$

\ref {Eq:ModspsInvStartUn}$_{k}$ $$\begin{aligned} 3_{k} \sum _{s \in \mathcal {S}_k} Q^{u}_{kst} - A^u_{kt0} = I^u_{kt0} \forall \, t\in \mathcal {T} \end{aligned}$$

\ref {Eq:ModspsInvBalUn}$_{k}$ $$\begin{aligned} 4_{k} \alpha _{h} \cdot I^u_{k,t-1,h-1} - A^u_{kth} = I^u_{kth} \forall \, t\in \mathcal {T},h\in \mathcal {H}\backslash \{0\} \end{aligned}$$

\ref {Eq:ModspsInvBalPro}$_{k}$ $$\begin{aligned} 5_{k} I^p_{k,t-1} + Q^o_{kt} + \sum _{s \in \mathcal {S}_k} Q^{p}_{kst} - A^p_{kt} = I^p_{kt} \forall \, t\in \mathcal {T} \end{aligned}$$

$$\begin{aligned} I^r_{k,t-1} + r_{kt} + \!\!\sum _{h=1}^{h^{\max }+1}\! (1- \alpha _{h}) \cdot I^u_{k,t-1,h-1} - \!\!\sum _{s \in \mathcal {S}_k}\!\! \left( Q^{p}_{kst} \!+\! Q^{u}_{kst} \right) \!=\! I^r_{kt} \quad \end{aligned}$$

\ref {Eq:ModspsInvBalR}$_{k}$ $$\begin{aligned} 6_{k} \forall \, t\in \mathcal {T} \end{aligned}$$

\ref {Eq:ModspsBigM}$_{k}$ $$\begin{aligned} 7_{k} Q^o_{kt} \le bigM_{kt} \cdot \gamma ^o_{kt} \forall \, t\in \mathcal {T} \end{aligned}$$

\ref {Eq:ModspsDefStag}$_{k}$ $$\begin{aligned} 12_{k} A^p_{kt},\,A^u_{kth} \in \mathbb {N}_0 \forall \, t\in \mathcal {T}, h \in \mathcal {H} \end{aligned}$$

\ref {Eq:ModspsDefInv}$_{k}$ $$\begin{aligned} 13_{k} I^p_{kt},\,I^r_{kt},\,I^u_{kth} \in \mathbb {N}_0 \forall \, t\in \mathcal {T}, h \in \mathcal {H} \end{aligned}$$

\ref {Eq:ModspsDefProRep}$_{k}$ $$\begin{aligned} 14_{k} Q^o_{kt},\,Q^{p}_{kst},\,Q^{u}_{kst} \in \mathbb {N}_0 \forall \, s \in \mathcal {S}_k,t\in \mathcal {T} \end{aligned}$$

\ref {Eq:ModspsBinBed}$_{k}$ $$\begin{aligned} 16_{k} \gamma ^o_{kt}\in \{0,1\} \forall \, t\in \mathcal {T} \end{aligned}$$

The objective function (27) minimizes the reduced costs of the newly derived procurement and reprocessing plan for the respective medical device *k*. The restrictions Eqs. ($2_{k}$) – ($7_{k}$), ($12_{k}$) – ($14_{k}$) and ($16_{k}$) are equivalent to the medical device-specific constraints of the PRPP.

Notably, the subproblem described is an extension of the single-item lot-sizing problem with perishable inventory presented by [19].

### Outline of the column generation approach

**Fig. 2:**
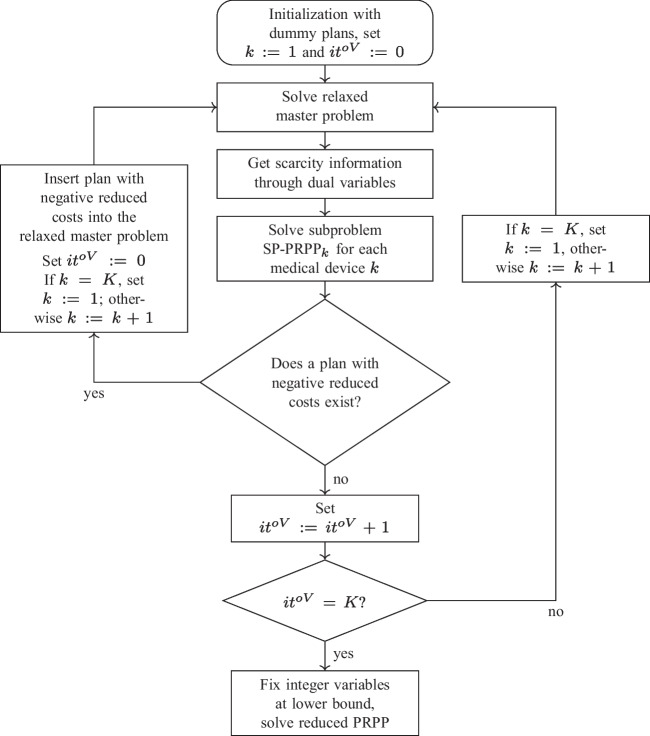
Flow chart of the CG approach for PRPP

A flow chart of the solution approach is presented in Fig. 2, cf. [31] . The column-generation approach is initialized with a dummy plan $n_0 \in \mathcal {N}_k$ for each medical device *k*. In this plan, all procurement and reprocessing quantities are fixed at 0; i.e., $\overline{Q}^{o(n_0)}_{kt} = 0$, $\overline{\gamma }^{o(n_0)}_{kt} = 0$ and $\overline{Q}^{p(n_0)}_{kst}=\overline{Q}^{u(n_0)}_{kst}=0$.

In addition, no medical device is stored either protected or unprotected; i.e., $\overline{I}^{p(n_0)}_{kt}=\overline{I}^{u(n_0)}_{kth}=0$. This means that these dummy plans do not consume any capacity in the MP-PRPP and are therefore formally feasible. However, these dummy plans cannot satisfy demand. Thus, to prevent the later selection of these dummy plans, prohibitively high costs are assigned to them.

After initialization, the iterative solution process begins. In each iteration, the LP relaxation of the MP-PRPP is solved to obtain updated dual variables for the next subproblem. A new procurement and reprocessing plan with negative reduced costs is determined by solving the medical device-specific subproblem SP-PRPP_k_ to optimality using CPLEX. If such a plan exists, it is included in the relaxed MP-PRPP. Since the inclusion of a new plan involves an improvement in the objective function value of the relaxed master problem, the counter of iterations $it^{oV}$ is reset to zero. Additionally, the relaxed master problem is solved once again, the subproblem of the medical device with the next higher index is considered, and a new iteration begins.

The inclusion of a new plan in the restricted MP-PRPP is accompanied by a change in the dual variables. Based on these updated dual variables, the next subproblem is solved. If no procurement and reprocessing plan with negative reduced costs can be identified, the counter $$it^{oV}$$ increases by 1 $$(it^{oV}:=it^{oV}+1)$$. If the counter $$it^{oV}$$ is less than the number *K* of medical devices, the medical device index is increased by 1 or reset to 1 (if $$k=K)$$ before the next iteration starts (right branch in Fig. 2). Otherwise, the counter $$it^{oV}$$ corresponds to the number *K* of medical devices. In this case, the procedure terminates because the objective function value of the restricted master problem has not improved for $$it^{oV}=K$$ iterations, and therefore, no further procurement and reprocessing plan for any of the medical devices exists, which reduces the objective function value of the master problem.

If a dummy plan $n_0$ has been selected for at least one medical device *k*, a feasible solution for the PRPP does not exist. Otherwise, the objective function value of the relaxed MP-PRPP constitutes a feasible lower bound. Notably, if both the selection variables $\vartheta _{kn}$ and the variables for the number of reprocessing operations $\chi ^r_{st}$ are integers after the termination of the column generation approach, this lower bound is also the optimal solution of the unrelaxed MP-PRPP and is thus also optimal for the PRPP.

However, it is usually the case that not all variables in the lower bound are integers. Nevertheless, the solution of the (relaxed) MP-PRPP can be used to solve the monolithic PRPP. Based on the procedure of [35], the corresponding binary variables $\gamma _{kt}^o$ of the procurement and reprocessing plans *n* for medical device *k* whose selection variables are integers, i.e., those that satisfy $\vartheta _{kn}=1$, are fixed in the solution of the relaxed master problem. Then, the reduced PRPP can be solved using CPLEX in a comparatively short amount of time under the assumption that the binary variables can be fixed for a large portion of medical devices. It should be noted that because these variables are fixed, a feasible solution for the reduced PRPP cannot be guaranteed, although such a solution exists for the monolithic problem. In addition, fixing these variables can prevent the global optimum from being found.

Furthermore, the variables $\chi _{st}^r$ from the final solution of the relaxed master problem can be rounded down and fixed according to (28) in the reduced PRPP; i.e.,

28 $$\begin{aligned} \chi _{st}^{r} = \big \lfloor \overline{\chi }_{st}^{r}\big \rfloor \forall \, s\in \mathcal {S}, t\in \mathcal {T}. \end{aligned}$$

Notably, this fixed number of reprocessing operations is always feasible with respect to capacity constraints (9), whereas rounding up may lead to a capacity overload. However, even with rounded down values, there is no guarantee that a feasible solution exists for the reduced PRPP. Thus, the fixation of the integer reprocessing variable $\chi _{st}^r$ can be removed after a short time limit. At best, a feasible initial solution will have been determined within this short time limit. After a second time limit to further improve the initial solution, this approach terminates with a procurement and reprocessing plan.

## Numerical analysis

### Design of test instances

For the numerical analysis, we define three problem classes (PCs) by varying the number of medical devices. A planning horizon of one week is assumed, and each day of the week is divided into three time slots, each with a length of eight hours. Thus, in total, 21 periods are considered. The maximum storage duration is limited to 48 hours for unprotected stored medical devices. Thus, seven storage periods $(h\in \lbrace 0,\dots ,6 \rbrace )$ must be taken into account; i.e., in storage period $h=5$, a medical device is stored for six periods and thus for 48 hours. Hence, in the subsequent storage period $h=6$, the medical device exceeds the maximum storage duration and is no longer sterile. Furthermore, three different time-temperature combinations (reprocessing types) are defined. Table 4 gives an overview of the PCs.

**Table 4:** Dimensions of problem classes

	Medical	Periods	Reprocessing	Maximum shelf
	devices $\vert \mathcal {K}\vert$	$\vert \mathcal {T}\vert$	types $\vert \mathcal {S}\vert$	life $\vert \mathcal {H}\vert$
PC I	20	21	3	7
PC II	40	21	3	7
PC III	100	21	3	7

As shown in Table 5, selected input parameters are systematically varied in each PC to analyze their impact on the numerical effort and solution quality. One test instance (TI) is generated for each parameter combination. Thus, in total, 216 test instances are examined for each problem class. The generation of test instances is described in more detail in [31] and Appendix A.

**Table 5:** Number of scenarios for the examined input parameters

Abbreviation	Symbol	$\#$ Characteristics
Start of planning [Monday or Thursday]	−	2
Coefficient of variation of demand	$vc^d$	2
Return portion	$\beta _k$	3
Capacity per reprocessing operation	$vol^{\max }$	3
Resource capacity in SSD	$c^{r}_t$	3
Storage capacity of unprotected stored medical devices	$c^{I^u}$	2

The numerical results presented in this paper are based on [31]. We implemented the model formulation of the PRPP and the solution approach based on column generation in the algebraic modeling system GAMS (Ver. 30.3.0). For the solution approach and the determination of reference values, we used CPLEX 12.10. The numerical study was conducted on the LENA cluster of the Leibniz Universität IT Services in Hannover using a single thread with a 2.40 GHz processor and 30 GB of RAM.

### Reference solutions

The CPLEX solver was able to determine a feasible solution for each test instance. Hereafter, the objective function value is called the CPLEX reference solution. Table 6 provides an overview of the solution quality of these reference solutions.

**Table 6:** Solution quality of CPLEX reference solutions

	$\text {TimLim}^{CPX}$	$\varnothing \text {IntGap}^{CPX}$	$\text {IntGap}^{CPX}_{\max }$	$\text {OptSol}^{CPX}$
PC I	3,600 s	0.36%	1.43%	2.78%
PC II	7,200 s	0.73%	1.92%	0.93%
PC III	14,400 s	0.82%	2.50%	0.00%

The computational time is limited for each test instance with respect to the problem class. This time limit is reported in column $\text {TimLim}^{CPX}$. For each PC, the average integrality gap is denoted by $\varnothing \text {IntGap}^{CPX}$, where the instance-specific integrality gap $\text {IntGap}_{TI}^{CPX}$ is derived as follows:

$$\begin{aligned} \text {IntGap}_{TI}^{CPX} = \frac{\text {ObjFun}^{CPX}_{TI}-\text {LowB}^{CPX}_{TI}}{\text {ObjFun}^{CPX}_{TI}} \cdot 100\%. \end{aligned}$$

For each TI, the parameter $\text {ObjFun}^{CPX}_{TI}$ describes the best known objective function value, and $\text {LowB}^{CPX}_{TI}$ denotes the best lower bound obtained by CPLEX within the given time limit. In column $\text {IntGap}^{CPX}_{\max }$, the maximum integrality gap is reported for each PC. Column $\text {OptSol}^{CPX}$ contains the proportions of optimally solved test instances.

As depicted in Table 6, the average integrality gap $\varnothing \text {IntGap}^{CPX}$ is relatively small, less than 1% for all PCs. In addition, the maximum integrality gap $\text {IntGap}^{CPX}_{\max }$ is rather small; it is 2.5% in PC III. Despite the large time limit, even in PC I, only a few test instances can be solved to the proven optimality. Furthermore, Table 6 shows that both the average and the maximum integrality gaps increase with increasing problem size.

### Results of the column generation-based solution approach

***Analysis of the lower bound***

In this section, the solution quality of the heuristic approach is presented and discussed. First, the quality of the lower bound obtained by column generation is evaluated in Table 7.

**Table 7:** Characteristics of the lower bound determined by column generation

	$\text {TimCG}$	$\varnothing \text {IntGap}^{CG}$	$\text {IntGap}^{CG}_{\max }$	$\varnothing |\mathcal {K}^{\text {fix}}|$	$\varnothing \text {IntGap}^{LP}$
PC I	123 s	0.86%	2.77%	68.29%	2.98%
PC II	268 s	1.52%	3.55%	78.06%	3.56%
PC III	528 s	1.20%	3.65%	86.44%	3.49%

A feasible lower bound was determined for each test instance. The entries in column $\text {TimCG}$ provide information regarding the average computational time of the CG approach. For each TI, the relative deviation ${\text {IntGap}^{CG}_{TI}}$ between the determined lower bound $\text {LowB}^{CG}_{TI}$ and the CPLEX reference solution ${\text {ObjFun}^{CPX}_{TI}}$ is calculated as follows:

$$\begin{aligned} \text {IntGap}_{TI}^{CG} = \frac{\text {ObjFun}^{CPX}_{TI}-\text {LowB}^{CG}_{TI}}{\text {ObjFun}^{CPX}_{TI}} \cdot 100 \% \end{aligned}$$

Hence, column $\varnothing \text {IntGap}^{CG}$ contains the average integrality gap for each PC. Column $\text {IntGap}^{CG}_{\max }$ shows the maximum deviation of the lower bound from the CPLEX reference solution. Furthermore, the proportion of medical devices with an integer solution in the lower bound, i.e., $\vartheta _{kn} =1$, is reported in column $\varnothing |\mathcal {K}^{\text {fix}}|$. Additionally, in column $\varnothing \text {IntGap}^{LP}$, the average deviation of the lower bound is compared to the LP relaxation of the PRPP, where

$$\begin{aligned} \text {IntGap}_{TI}^{LP} = \frac{\text {ObjFun}^{CPX}_{TI}-\text {ObjFun}^{LP}_{TI}}{\text {ObjFun}^{CPX}_{TI}} \cdot 100\%. \end{aligned}$$

For each TI, the optimal solution of the LP relaxation is denoted by $\text {ObjFun}^{LP}_{TI}$.

**Fig. 3:**
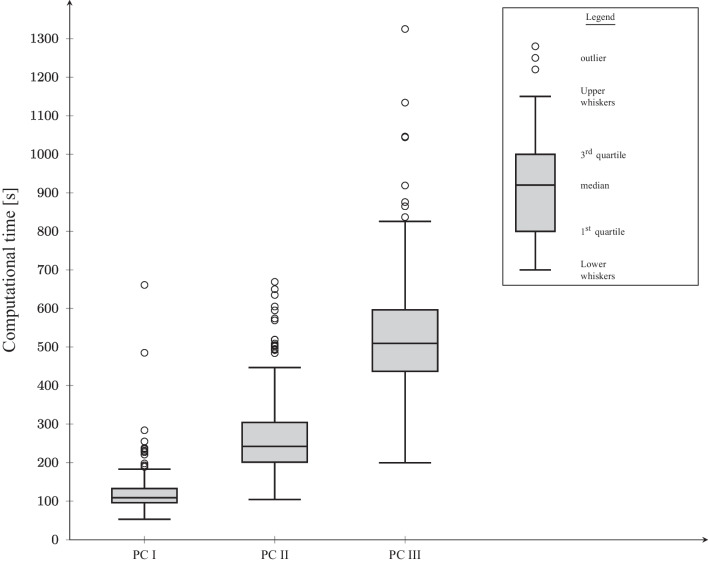
Spread of the computational times of the CG approach

As expected, the computational time required to determine the lower bound increases with the number of medical devices. Nevertheless, even in PC III, the computational time is less than nine minutes on average. However, within each PC, the computational time of individual test instances deviates substantially from the average. The boxplot diagram in Fig. 3 gives a better impression of the observed computational times for each PC. Notably, the box plot whiskers describe the data points that are limited to a maximum of 1.5 times the interquartile range.

For 75% of the instances in PC I, the column generation approach terminates in less than 133 seconds. Furthermore, the computational time for 75% of the instances does not exceed 300 seconds in the case of PC II or 600 seconds in the case of PC III. Individual outliers can be observed for all PCs, deviating significantly from the average computational time. However, the computational time of these outliers is less than 2.5 times the average computational time. With one exception, all outliers belong to parameter constellations in which the planning horizon begins on Thursday. In these test instances, the reprocessing capacity on the weekend is used to increase the inventory of protected stored medical devices. Thus, the capacity for protected storage also becomes scarce in these periods. This scarcity substantially influences the structure of the generated procurement and reprocessing plans since more plans must be generated during column generation. On average, test instances whose planning horizon begins on a Thursday require 1.3 times the computing time of TIs whose planning begins on a Monday. Notably, the capacity of protected storage is not fully exhausted in any period if planning begins on a Monday.

The average integrality gap $\varnothing \text {IntGap}^{CG}$ is lower than 1.6% for all problem classes. Furthermore, the lower bound obtained by column generation is obviously tighter than the LP relaxation. The maximum deviation $\text {IntGap}^{CG}_{\max }$ does not exceed 4.7%, which also indicates the high quality of the lower bound obtained by column generation. The portion $\varnothing |\mathcal {K}^{\text {fix}}|$ of medical devices for which exactly one order quantity and reprocessing plan are selected in the final solution of the relaxed master problem $(\vartheta _{kn} =1)$ increases from approximately 68% in PC I to more than 86% in PC III.

***Analysis of the upper bound***

Based on the generated lower bound, a feasible solution is determined for each TI. The solution quality of these upper bounds is analyzed below. The numerical results are summarized in Table 8.

**Table 8:** Characteristics of the upper bound obtained by column generation

	$\text {TimLim}^{UB}$	$\text {FeasSol}^{UB}_{10\%}$	$\varnothing \text {Dev}^{UB,CPX}$	$\varnothing \text {IntGap}^{UB,LB}$	$\text {BetSol}^{UB}$
PC I	180s	97.22%	0.23%	1.10%	10.18%
PC II	300s	96.19%	0.39%	1.95%	11.57%
PC III	600s	99.54%	0.75%	1.98%	14.81%

Although a large number of binary variables can be fixed after the column generation approach terminates, the reduced PRPP still has a large number of integer decision variables. Therefore, a PC-specific time limit is also used for the generation of a feasible solution. This time limit is provided in column $\text {TimLim}^{UB}$. To quickly determine a (first) feasible upper bound, the number of reprocessing operations $\chi ^r_{st}$ of type *s* obtained from the final solution of the relaxed master problem is rounded down to the next integer and initially fixed. This fixation reduces the solution space and usually accelerates optimization. However, the fixation of the variable $\chi ^r_{st}$ is removed after a maximum of 10% of the time limit specified in column $\text {TimLim}^{UB}$. Column $\text {FeasSol}^{UB}_{10\%}$ reports the proportion of instances with feasible solutions that were already found after 10% of the time limit had passed.

The remaining computational time is used to improve this initial solution; however, after that, all binary procurement variables are continuously fixed. Otherwise, if no initial solution is found, the previous fixation of variables $\chi ^r_{st}$ is removed, and the reduced PRPP with only fixed binary variables is solved. Using this approach, a feasible upper bound $\text {UpB}_{TI}$, i.e., a feasible solution, is found for each TI. The deviation of this upper bound from the CPLEX reference solution $\text {ObjFun}_{TI}^{CPX}$ is calculated as follows:

$$\begin{aligned} \text {DEV}^{UB,CPX}_{TI} = \frac{\text {UpB}_{TI}-\text {ObjFun}_{TI}^{CPX}}{\text {ObjFun}_{TI}^{CPX}} \cdot 100\%. \end{aligned}$$

In column $\varnothing \text {DEV}^{UB,CPX}$, the PC-specific deviation is provided. Column $\text {BetSol}^{UB}$ reports the proportion of test instances with an upper bound $\text {UpB}_{TI}$ that is at least as good or better than the CPLEX reference value $\text {ObjFun}_{TI}^{CPX}$. Furthermore, $\varnothing \text {IntGap}^{UB,LB}$ describes the average deviation between the upper and lower bounds determined by column generation and can be derived for each instance as follows:

$$\begin{aligned} \text {IntGap}^{UB,LB}_{TI} = \frac{\text {UpB}_{TI} - \text {LowB}_{TI}^{CG}}{\text {LowB}_{TI}^{CG}} \cdot 100\%. \end{aligned}$$

**Table 9:** Comparison of the reference solutions with a reduced time limit

	CPLEX solver			
	$\text {FeasSol}^{CPX}_{\text {red}}$	$\varnothing \text {IntGap}^{CPX}_{\text {red}}$	$\text {IntGap}^{CPX}_{\max ,\text {red}}$	$\text {TimLim}_{\text {red}}^{CPX}$
PC I	99.07%	0.52%	1.98%	480 s
PC II	98.61%	1.04%	2.70%	900 s
PC III	87.04%	1.22%	25.17%	1800 s
	Solution approach			
	$\text {FeasSol}^{UB}_{\text {red}}$	$\varnothing \text {Dev}^{UB,CPX}_{\text {red}}$	$\text {Dec}^{UB,CPX}_{\max ,\text {red}}$	$\text {BetSol}^{UB}_{\text {red}}$
PC I	100.00%	0.13%	1.37%	30.55%
PC II	100.00%	0.14%	3.78%	35.65%
PC III	100.00%	0.32%	16.15%	27.78%

For each test instance, the time limit provided to determine an upper bound was fully exhausted. For more than 96% of the test instances, a feasible initial solution was determined based on the fixed number of reprocessing operations. The average computational time required to determine this initial solution for PRPP based on this fixation was less than one second in the case of PC I, less than five seconds for PC II and less than 21 seconds for PC III.

When the time limit $\text {TimLim}^{UB}$ is reached, on average, the upper bound deviates by less than 1% from the CPLEX reference solution for all PCs. This emphasizes the high solution quality. This deviation tends to increase slightly in all PCs if the reprocessing capacity $c^r_t$ is decreased. The integrality gap $\varnothing \text {IntGap}^{UB,LB}$ is less than 2% even in PC III and is therefore very small.

In addition, for more than 10% of the instances, a solution was found that is at least as good or better than the CPLEX reference solution. The average computational effort needed to determine this solution amounts to less than 10% of the provided time limit for CPLEX to generate a reference solution. However, it is possible that CPLEX will find a good solution at the very beginning of the optimization process and use the majority of the computational effort to prove optimality or to raise the lower bound. Thus, the investigations below should allow a fairer comparison between CPLEX and the proposed solution approach.

***Reference solutions with a comparable computational time***

To define an admissible time limit for CPLEX to determine a reference solution for each PC, the average computational time $\text {TimCG}$ for generating the lower bound (see Table 7) was first rounded up to the next full minute. Then, the given time limit $\text {TimLim}^{UB}$ for generating an upper bound, according to Table 8, was added. Since the computational effort for determining the lower bound varies depending on the instance, the sum of the run times was multiplied by a factor of 1.5. The resulting PC-specific time limit is shown in column $\text {TimLim}^{CPX}_{\text {red}}$ in Table 9. Notably, within this new time limit, the solution approach based on column generation terminates earlier in more than 98% of the instances. This time limit corresponds to about an eighth of the previous time limit that was given to CPLEX for determining the reference solutions in Section 6.2.

Column $\text {FeasSol}^{CPX}_{\text {red}}$ in the upper part of Table 9 indicates the proportion of instances for which CPLEX found a feasible solution within the reduced time limit. For each test instance, the integrality gap $\text {IntGap}^{CPX}_{\text {red}}$ is determined. Column $\varnothing \text {IntGap}^{CPX}_{\text {red}}$ shows the average integrality gap of each PC. In column $\text {IntGap}^{CPX}_{\max ,\text {red}}$, the maximum integrality gap is given.

The results of the proposed solution approach are described in the lower part of Table 9. The structure of this table is similar to the structure of Table 8. The additional column $\text {Dev}^{UB,CPX}_{\max ,\text {red}}$ indicates the maximum deviation of the upper bound from the new reference solution. Column $\text {BetSol}^{UB}_{\text {red}}$ again gives the proportion of instances for which the objective function value is at least as good or better than the new reference solution. Notably, for the comparison, only test instances for which CPLEX was able to find a feasible solution within the specified new time limit were examined.

Within this new time limit, CPLEX was not able to find a feasible solution for all TIs. The portion of TIs for which no feasible solution was found within this time limit increases with the number of medical devices. While CPLEX found a feasible solution for more than 98% of instances in PC I and II, CPLEX failed to determine a feasible solution for 28 of 216 test instances in PC III. Our solution approach, on the other hand, was able to generate a feasible solution with less computational effort for all TIs.

For the TIs with a feasible solution, the average integrality gap $\varnothing \text {IntGap}^{CPX}_{\text {red}}$ is relatively small for all PCs. Integrality gaps higher than 25% are only found for individual outliers. It is worth mentioning that the proposed solution approach determines feasible solutions of high quality that deviate by less than 0.4% from the reference solution on average, even for the largest PC. For approximately 30% of the test instances, the solution approach terminates with a feasible solution that is at least as good as or better than the new CPLEX reference solution.

### Comparison to first–come-first-served simulation

To provide a baseline for further comparison, we follow the idea of [10] to use a first-come–first-served (FCFS) approach for reprocessing medical devices. Such a FCFS approach is quite common in SSDs. Therefore, we implemented a simulation approach which is guided by the FCFS principle.

The procedure for each period *t* can be described as follows:

- At the beginning of each period, the net demand is derived for each medical device *k* by taking the current inventory into account. Notably, the inventory is withdrawn according to the FIFO principle, where medical devices in unprotected storage are preferred.
- Based on the period-specific returns $r_{kt}$ for all medical devices, the returns are randomly arranged unit by unit and stored in serial order. Following this order, we try to assign all returned units to a reprocessing operation via the following procedure:
  1. If at least one reprocessing operation with sufficient capacity is scheduled, the current medical device unit is assigned to the reprocessing operation with the fastest reprocessing time,
    i. if there is unsatisfied demand for this medical device, this unit is taken directly for demand satisfaction;
    ii. else if the net demand has been fulfilled and enough storage capacity is left, this unit is stored (unprotected storage preferred);
    iii. otherwise the current unit is skipped.
- Regardless of which of the above cases i. to iii. was selected, inventory levels are set according to inventory balance constraints (2) to (6).
  2. else if the capacity of the reprocessing resource is sufficient and either net demand for the current medical device is unsatisfied or storage capacity is left, an additional reprocessing operation is scheduled (preferably with the fastest reprocessing time, that can reprocess the current medial device unit) and go to 1;
  3. otherwise the current unit is skipped.
- If the current medical device unit has no successor, the assignment for the current period ends. Demand that is not met by neither inventory nor reprocessing must be procured. Medical device units that have been skipped are considered first in the following period $t+1$.

We passed the obtained simulation results to the PRPP, where we fixed the variable values, i.e., variables (12) – (16), to verify feasibility of the procurement and reprocessing plans.

Next, we analyze the results of the simulation from a managerial perspective; therefore, six TI of PC 1 were selected for the simulation that differ in available resource $c^r$ (low, medium, and high) and storage capacity $c^{I^u}$ (low, high). For each instance, 1000 replications were performed, and the mean values were determined. These mean values are compared with the corresponding solution of the monolithic PRPP. Our evaluation concentrates on the obtained procurement and reprocessing plans.

An instance-specific cost comparison is provided in Table 10.

**Table 10:** Cost comparison between PRPP and simulation study

Case ($c^{I^u}$/$c^r$)	Obj. PRPP	Mean Value	Mean Deviation
		incl. 95% confidence interval	
Case 1 (high/low)	95,793.5	$370,727.3 \pm 190.6$	$387.01\% \pm 0.20\%$
Case 2 (high/medium)	86,921.5	$335,693.5 \pm 265.7$	$386.20\% \pm 0.31\%$
Case 3 (high/high)	80,002.5	$287,354.9 \pm 529.3$	$359.19\% \pm 0.66\%$
Case 4 (low/low)	95,868.5	$363,698.3 \pm 159.1$	$379.37\% \pm 0.17\%$
Case 5 (low/medium)	87,482.5	$332,738.4 \pm 203.7$	$380.35\% \pm 0.23\%$
Case 6 (low/high)	80,937.5	$294,942.2 \pm 424.5$	$364.41\% \pm 0.52\%$

The results of the simulation study show that the mean values of the total costs are on average 3.76 times higher than the objective function value of the monolithic PRPP. The 95% confidence interval is rather small. In the case of low resource capacity, the total cost is higher, but the 95% confidence interval is smaller compared to the case of high resource capacity.

While the total number of reprocessing operations scheduled during the simulation is approximately 17.5% lower than that of the solution of the PRPP, the simulated capacity consumption is only slightly lower with a deviation between 1.3% and 4.5%. This can be explained by the fact that slow reprocessing operations were scheduled more often than fast reprocessing operations. In contrast, fast reprocessing operations are prioritized during optimization to increase the number of reprocessed devices and thus avoid procurement. During the simulation, medical devices that can be reprocessed by a faster reprocessing operation are also integrated into slow reprocessing operations. After optimization, this case is rarely observed. Scheduling a larger number of slow reprocessing operations means that the scarce capacity of the decontamination resource is used up more quickly. As a result, orders must be placed myopically and therefore much more frequently. For example, we found that the simulated solutions exceeded both the orders placed and the quantities procured by a factor of four.

The simulation results also show that some medical devices exceeded their maximum shelf life after reprocessing. Therefore, reprocessing of this device was unnecessary. This happens very rarely for medical devices with regular high demand. However, for products with sporadic demand, we observed that products exceeded shelf-life up to twice a week. This results in unnecessary reprocessing costs as well as unnecessary capacity consumption. In the optimized solution, shelf-life exceedance does not occur after reprocessing.

## Conclusion and outlook

In this paper, we presented a new model formulation for integrated procurement and reprocessing planning of reusable medical devices with a limited shelf life. Based on a surgery schedule with a planning horizon of one week, the objective of the PRPP is to determine a feasible procurement and reprocessing plan for the SSD in a hospital. To solve the PRPP, a solution approach based on the principle of Dantzig-Wolfe decomposition and column generation was presented. As part of an extensive numerical study, the solution quality of this approach was examined. The integrality gaps of the lower bound obtained by column generation are very close to the optimal solution. The applied solution approach yields a high solution quality, and the solutions are very close to the CPLEX reference values. However, the numerical effort is substantially reduced. When the time limit was reduced, unlike the presented solution approach, CPLEX was not able to find a feasible solution for all TIs.

Future research will address different model extensions. Regarding the procurement of medical devices, minimum order quantities or quantity discounts as well as the selection of external suppliers can be integrated into the model formulation. Regarding reprocessing, the model formulation can be extended to take several parallel resources into account to reduce bottlenecks in reprocessing operations. However, if these resources do not differ, this extension involves many redundant or symmetric solutions. Thus, additional constraints are required to avoid symmetries. Hence, an adaptation of the solution approach is likely needed, since a smaller number of integer variables can be expected to appear in the final solution of the relaxed master problem. Additionally, the subproblems can be solved heuristically to reduce the numerical effort. However, to guarantee a feasible lower bound, the MILP solver is only required if the heuristic cannot identify any further plan with negative reduced costs. This approach can be extended to an exact branch &price approach, where column generation occurs in each node of the search tree.

Furthermore, the demand for medical devices is derived directly from the surgery schedule. Thus, the demand can be assumed to be deterministic. It is also assumed that the surgery schedule is executed without any changes, such that the use of medical devices always takes place in the specified period. However, due to short-term staff absences or urgent emergency surgeries, deviations from the schedule may occur, leading to uncertainty in both demand and return data. To cope with these uncertainties, e.g. sample average approaches can be used, cf. [16] and [18], to determine a robust procurement and reprocessing plan. Depending on the condition of the used medical device, it may be necessary to go through the reprocessing cycle several times. As a result, the affected medical device may not be used immediately after reprocessing. Therefore, further research should also investigate the robustness of the determined procurement and reprocessing plan. In addition, the simultaneous consideration of stochastic demand and return data should be considered.

## Figures and Tables

**Fig. 4:**
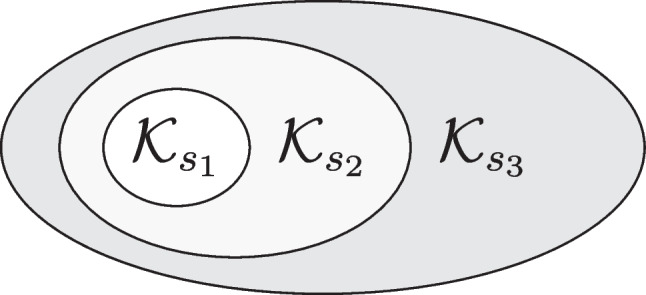
Assignment of medical devices to reprocessing types $\mathcal {K}_s$

## Data Availability

The test instances of this study are available from the authors upon request.

## A Detailed description of the test instances

For the generation of test instances, see also [31], we assume a hospital in which surgeries of elective patients take place in two shifts from Monday to Friday. Therefore, the demand for medical devices is positive in at most ten periods. Hereafter, these periods are called surgical periods $t'\in \mathcal {T}$. In Table 11, the surgical periods are highlighted in bold.

**Table 11:** Surgical periods (start of planning: Monday)

	Mon.	Tue.	Wed.	Thr.	Fri.	Sat.	Sun.
Time slot 1	**1**	**4**	**7**	**10**	**13**	16	19
Time slot 2	**2**	**5**	**8**	**11**	**14**	17	20
Time slot 3	3	6	9	12	15	18	21

The SSD, on the other hand, also operates in two shifts (time slots 1 and 2), but these shifts are from Monday to Sunday. In an alternative scenario, the planning horizon begins on Thursday to vary the position of the surgical periods. Thus, the third time slot on Wednesday represents the last period of the planning horizon in the second scenario.

Using an ABC analysis, [32] examines both the demand and the turnover ratio of medical devices. They point out that a relatively small portion of medical devices account for most of the demand. In contrast, the majority of medical devices possess an irregular demand with small quantities. Following this observation, an XYZ analysis is used to assign medical devices according to their demand and turnover ratio to one of three classes $\kappa \in \lbrace X, Y, Z\rbrace$. The characteristics associated with each class are summarized in Table 12.

**Table 12:** Class-specific characteristics of medical devices

	surgical periods	Proportion	Distribution of
	with $d_{kt'}>0$		demand
Class *X*	10 of 10	$\Omega ^X = 20\%$	$d_{kt'}^X\sim \mathcal {N}(\mu _k^X,\sigma _k^2)$
Class *Y*	4 of 10	$\Omega ^Y =30 \%$	$d_{kt'}^Y\sim \mathcal {N}(\mu _k^Y,\sigma _k^2)$
Class *Z*	1 of 10	$\Omega ^Z =50 \%$	$d_{kt'}^Z\sim \mathcal {N}(\mu _k^Z,\sigma _k^2)$

Class *X* includes regularly required basic instruments. Medical devices in this class are required in each surgical period; i.e., the demand is comparatively high. Department-specific instruments constitute Class *Y*, while Class *Z* includes special surgery-specific instruments. The medical devices in Class *Z* are therefore required less often. Thus, these medical devices are only required in one out of ten surgical periods. For example, in PC I, ten different medical devices belong to Class *Z*. Each of these medical devices is required in a different surgical period. The demand for medical devices in Classes *Y* and  *Z* is evenly distributed over the surgical periods.

The expected value $\mu _k^\kappa$ of the demand for medical device *k* is determined by a uniform distribution in a given interval depending on the assigned $\kappa$. $\mu _k^X\in [150,250]$, $\mu _k^Y\in [40,60]$ and $\mu _k^Z\in [4,6]$ apply to these integer intervals, so the class-specific expected values of the demand are $\mu ^X=200$, $\mu ^Y=50$ and $\mu ^Z=5$. Using a (truncated) normal distribution, the demand $d_{kt'}^{\kappa }$ is then generated in the surgical periods $t'$ and rounded to the next integer. The standard deviation is $\sigma _k=\frac{1}{10}\cdot \mu _k^\kappa$, so the coefficient of variation $vc^d$ is $\frac{1}{10}$. In an alternative scenario, the standard deviation is $\sigma _k=\frac{3}{10}\cdot \mu _k^\kappa$; thus, the coefficient of variation is $\frac{3}{10}$. Notably, the normal distribution theoretically enables a negative realization of the demand. These realizations are set to 0.

For the proportion of returned medical devices, three different values are defined in Classes *X* and *Y*, namely, $\big (\beta _k^X, \beta _k^Y \in \lbrace 98\%, 95\%, 90\% \rbrace \big )$. For medical devices of Class *Z*, on the other hand, it is assumed that due to the low turnover ratio and the associated lower level of wear and tear, no medical devices need to be disposed of $(\beta _k^Z=1)$. The return $r_{kt}$ of medical device *k* in period *t* is determined by $r_{kt} = \lfloor \beta _k^\kappa \cdot d_{k,t-2} \rfloor$. The return quantities in the first and second periods of the planning horizon depend on the class, and $r_{k1}=r_{k2}=\lfloor \beta _k^\kappa \cdot \mu _{d^{\kappa }} \rfloor$.

The fixed procurement costs $oc^o_k$ are normalized to $oc^o_k=250$ for each medical device *k*. The variable costs $pc^o_k$ for one unit of medical device *k* follow a uniform distribution in the real-valued interval $pc^o_k \in [25,125]$ and are then rounded off in steps of 0.5.

The medical devices are assigned to the processing types based on the $A_0$ concept explained in Section 2. Due to the temperature sensitivity of some medical devices, it can be expected that only a comparatively small portion of medical devices can be reprocessed at very high temperatures. Since compatibility restrictions only exist for temperatures that are too high, all medical devices can be reprocessed using the reprocessing type with the lowest temperature. The diagram in Fig. 4 illustrates this relationship.

The medical devices are assigned to the subset $\mathcal {K}_{s_1}$ with probability $P[k \in \mathcal {K}_{s_1}]=\frac{1}{3}$. If an assignment is made, these medical devices can also use reprocessing types $s_2$ and $s_3$. Otherwise, the remaining medical devices are assigned to the subset $\mathcal {K}_{s_2}$ with probability $\frac{1}{2}$; i.e., $P[k \in \mathcal {K}_{s_2}]= \frac{1}{3} + \frac{2}{3}\cdot \frac{1}{2}=\frac{ 2}{3}$. The sterilization type $s_3$ can be used by all medical devices, i.e., $(P[k \in \mathcal {K}_{s_3}]=1)$.

The variable packaging costs for unprotected and protected storage are $pc_{k}^u=1$ or $pc_{k}^p=2$ per unit of medical device *k*. The reprocessing costs $sc_s^r$ per operation of type *s* and the associated duration $ts_s^r$ are also based on the $A_0$ concept. Consequently, the reprocessing time decreases with increasing temperature. Furthermore, reprocessing types with high process temperatures incur higher costs due to the higher energy requirement. Table 13 shows the reprocessing costs $sc_s^r$ and the duration $ts_s^r$ per operation of type *s*.

The space requirement $vol_k$ for reprocessing one unit of medical device *k* is uniformly distributed in the interval $vol_k\in [5,15]$. The average space requirement $\overline{vol}^{\kappa }$ is determined for each class $\kappa$. The capacity $vol^{\max }$ per reprocessing operation depends on the PC. In PC I, $\overline{vol}^{\max }=250$ capacity units per operation are available, while in PC II or PC III, 500 or 1, 000 capacity units per operation are available. The capacity per reprocessing operation is varied in each problem class by the factor $vg\in \lbrace \nicefrac {4}{5},1,\nicefrac {6}{5} \rbrace$.

The capacity depends on the PC and the regular reprocessing capacity $\overline{vol}^{\max }$. From Monday to Friday, there are

$$\begin{aligned} c^r_{t}= \bigg \lceil \big (1-c^{em}\big ) \cdot \frac{|\mathcal {K} |\cdot \big (\sum _{ \kappa }\Omega ^{\kappa } \cdot \mu ^{\kappa } \cdot \overline{vol}^{\kappa } \big ) \cdot \sum _{s\in \mathcal {S}} ts_s^ r}{2\cdot |\mathcal {S} |\cdot \overline{vol}^{\max }} \bigg \rceil \end{aligned}$$

capacity units available in the first and second time slots, while this capacity is halved on weekends. Furthermore, there is no capacity available in the third time slot. Similar to the surgery schedule, the capacity for reprocessing medical devices for emergency patients is reserved in the SSD in the amount of $c^{\text {em}}\in [0,1]$. This factor is examined in three different forms $\big (c^{\text {em}}\in \lbrace 20\%, 10\%, 0\% \rbrace \big )$.

**Table 13:** Reprocessing type-specific costs and duration per reprocessing operation

	Costs $sc_s^r$	Duration $ts_s^r$
Reprocessing type ${s_1}$	200	25
Reprocessing type ${s_2}$	100	40
Reprocessing type ${s_3}$	50	60

The PC-specific storage capacity for protected medical devices is limited in each period to

$$\begin{aligned} c^{I^p}= \Big \lceil \tfrac{1}{2}\cdot |\mathcal {K} |\cdot \Big (\sum _{\kappa }\Omega ^{\kappa } \cdot \mu ^{\kappa } \cdot \overline{vol}^{\kappa }\Big ) \Big \rceil \qquad \forall \, t \in \mathcal {T}. \end{aligned}$$

Depending on this size, two forms $\left( c^{I^u}\in \big \lbrace \big \lceil \nicefrac {1}{3}\cdot c^{I^p}\big \rceil , \big \lceil \nicefrac {1}{2}\cdot \right.$ $\left. c^{I^p}\big \rceil \big \rbrace \right)$ are analyzed. $I^u_{k01}=I^u_{k02}=\big \lfloor \nicefrac {1}{10}\cdot \mu ^{\kappa } \big \rfloor$ applies to the initial inventory of unprotected and protected stored medical devices, and $I^p_{k0}=\big \lfloor \frac{1}{3}\cdot \mu ^{\kappa }\big \rfloor$ applies to each medical device *k*. The initial inventory of used medical devices is $I^r_{k0}=\big \lfloor \nicefrac {1}{3}\cdot \mu ^{\kappa }\big \rfloor$.
